# Heme oxygenase and carbon monoxide protect from muscle dystrophy

**DOI:** 10.1186/s13395-016-0114-6

**Published:** 2016-11-28

**Authors:** Mun Chun Chan, Olivia Ziegler, Laura Liu, Glenn C. Rowe, Saumya Das, Leo E. Otterbein, Zoltan Arany

**Affiliations:** 1 Cardiovascular Institute, Beth Israel Deaconess Medical Center, Boston, MA USA; 2 Current address: Cardiovascular Institute, Massachusetts General Hospital, Boston, MA USA; 3 Present Address: Department of Medicine, University of Alabama at Birmingham, Birmingham, AL USA; 4 Department of Surgery, Beth Israel Deaconess Medical Center, Boston, MA USA; 5 Cardiovascular Institute and Institute Diabetes Obesity and Metabolism, Smilow Center for Translational Research, Perelman School of Medicine, University of Pennsylvania, 11th floor, 3400 Civic Blvd, Philadelphia, 19104 PA USA

**Keywords:** PGC-1α, mdx, Duchenne muscle dystrophy, Heme oxygenase, HO-1, Carbon monoxide

## Abstract

**Background:**

Duchenne muscle dystrophy (DMD) is one of the most common lethal genetic diseases of children worldwide and is 100% fatal. Steroids, the only therapy currently available, are marred by poor efficacy and a high side-effect profile. New therapeutic approaches are urgently needed.

**Methods:**

Here, we leverage PGC-1α, a powerful transcriptional coactivator known to protect against dystrophy in the *mdx* murine model of DMD, to search for novel mechanisms of protection against dystrophy.

**Results:**

We identify heme oxygenase-1 (HO-1) as a potential novel target for the treatment of DMD. Expression of HO-1 is blunted in the muscles from the *mdx* murine model of DMD, and further reduction of HO-1 by genetic haploinsufficiency worsens muscle damage in *mdx* mice. Conversely, induction of HO-1 pharmacologically protects against muscle damage. Mechanistically, HO-1 degrades heme into biliverdin, releasing in the process ferrous iron and carbon monoxide (CO). We show that exposure to a safe low dose of CO protects against muscle damage in *mdx* mice, as does pharmacological treatment with CO-releasing molecules.

**Conclusions:**

These data identify HO-1 and CO as novel therapeutic agents for the treatment of DMD. Safety profiles and clinical testing of inhaled CO already exist, underscoring the translational potential of these observations.

## Background

Duchenne muscular dystrophy (DMD) is a common (1 in 3500 boys), X-linked recessive, inexorably lethal disease characterized by acute phases of muscular degeneration and insufficient compensatory regeneration [1]. Diagnosis occurs early in life from noticeable walking difficulty. The disease is 100% fatal. Death occurs by the early twenties from respiratory or cardiac failure caused by damage to the heart and diaphragm muscles. There is no known cure for DMD. DMD is caused by mutations in the *dystrophin* (*DMD*) gene that lead to loss of functional protein. Dystrophin protein is a part of the large dystrophin-glycoprotein complex that links the inner cytoskeleton of muscle cells with the extracellular basal lamina. Loss of dystrophin leads to dysfunction and necrosis of mature muscle cells including an increase in membrane damage from mechanical or hypo-osmotic stress and decreased function of plasma membrane proteins including neuronal nitric oxide synthase (nNOS) [1–4]. Though the genetic cause of DMD is known, how dystrophin mutations lead to muscle damage is incompletely understood, nor how to reverse the process. Significant efforts have focused recently on achieving total cure for DMD, including approaches such as exon skipping and most recently CRISPR-mediated genome editing [5–7], but significant hurdles persist. A complementary approach, which may be more imminently tractable, is the possibility of altering the natural course of the disease, via inhibiting degeneration and/or stimulating regeneration of muscle cells.

The transcriptional coactivator peroxisome proliferator-activated receptor gamma coactivator 1-α (PGC-1α) is a powerful activator of broad metabolic programs in various tissues [8–10]. In the skeletal muscle, PGC-1α strongly induces mitochondrial biogenesis, resulting in mice resistant to fatigue [11, 12]. A number of years ago, crossing mice that transgenically express PGC-1α in the skeletal muscle into the dystrophin-deficient mice (*mdx*) background, a well-established murine model of DMD, revealed impressive protection against dystrophy [13]. The protection has widely been presumed to result from the strong PGC-1α-mediated induction of neuromuscular junction genes and, in particular of utrophin, a homolog of dystrophin that is known to be protective in DMD models. However, we recently generated mice with muscle-specific expression of peroxisome proliferator-activated receptor gamma coactivator 1-β (PGC-1β), a homolog of PGC-1α, and found that PGC-1β did *not* induce neuromuscular junction (NMJ) genes or utrophin itself and yet was equally protective against dystrophy in the *mdx* model [14]. This suggested that utrophin was not the key mediator of protection in the PGC-1α transgenic mice. We formally tested this notion by crossing the PGC-1α-expressing mice into an *mdx*/utrophin^−/−^ background, which at baseline reveal much more pronounced dystrophy, including blunted growth, kyphosis, and early lethality. PGC-1α significantly improved the dystrophic phenotype of these mice, including early lethality [14]. PGC-1α thus potently protects against dystrophy, but not via utrophin.

How does PGC-1α protect against muscle dystrophy, if not via utrophin? We investigate here two possibilities: induction of vasculature and induction of *Hmox1*, the gene encoding for heme oxygenase (HO-1).

## Methods

### Animals

All animal experiments were performed according to the procedures approved by the Beth Israel Deaconess Medical Center’s Institutional Animal Care and Use Committee. All experimental animals used were male. C57BL/10ScSn-*Dmd* ^mdx^/J mice (*mdx* mice) were obtained from Jackson Laboratories. These mice were crossed with muscle-specific PGC-1α transgenic mice (MCK-PGC-1α), described previously [11]; muscle-specific PGC-1β transgenic mice (MCK-PGC-1β) described previously [15]; or mice with inducible PGC-1α (Ind_PGC-1α) that possess the muscle-specific tetracycline-dependent activator (MCK-tTA), described previously [16], and the PGC-1α coding region under control of the tet-response element promoter (TRE-PGC-1α) transgenic genes, described previously [16]. All of these mice were in C57BL/6 background; progeny were therefore in mixed C57BL/6 and C57BL/10 background. For all experiments, littermates were used as controls, and male animals were evaluated. *mdx*, *mdx*/MCK-PGC-1α, *mdx*/MCK-PGC-1β, and *mdx*/Ind_PGC-1α were sacrificed at 9 weeks of age (*n* = 5 transgenic, 5 controls). Male HMOX^+/−^ mice (FVB.129S2(B6)-Hmox1^tm1Poss^/J) were obtained from Jackson Laboratories (JAX008311) and crossed with female C57BL/10ScSn-*Dmd* ^mdx^/J mice. HMOX^+/−^/*mdx* animals are therefore in mixed background, and, again, litter-mates were strictly used for control (*n* = 8 experimental; 8 control). Ind_tsFLT mice, engineered to express in skeletal muscle inducible sFlt1 in a tet-OFF fashion, have been used by us previously [17] and were generated by crossing transgenic mice expressing tet-inducible sFlt1, previously described [18], with transgenic mice expressing muscle-specific expression of the tTA transactivator, as above. All doxycycline-responsive mice are in a tet-OFF system, and mice were maintained on tetracycline chow throughout pregnancy and development, and switched to regular chow at 5 weeks of age, followed by evaluation and harvesting at 9 weeks of age. Littermate controls were used for all experiments.

Protoporphyrin IX cobalt chloride (CoPP) (Sigma-Aldrich) was dissolved in NaOH (0.2 M) and then adjusted to pH 7.6 with HCl. 10 mg/kg/day of dissolved CoPP was injected three times a week intra-peritoneally into mdx male mice from 5 to 9 weeks of age (*n* = 10 CoPP; 7 control). Age-matched mdx control mice were injected with HCl-neutralized NaOH (pH 7.6). Male mdx mice were exposed to carbon monoxide (CO) gas at a concentration of 250 ppm for 1 h a day, 5 days a week, for 4 weeks total from 5 to 9 weeks of age. Mice were then sacrificed. Control mdx mice were placed in an equivalent-sized plexiglass box for 1 h a day (*n* = 8 CO, 8 control). Male mdx mice were injected with 2.5 mg/kg/day of CO-releasing molecule A1 (CORM-A1) from 5 to 9 weeks of age. CORM-A1 was dissolved 5 min before injection freshly each day. Inactivated CORM-A1 was obtained by dissolving CORM-A1 and then piping nitrogen through the solution for 15 min to activate release of all CO. Inactivated CORM-A1 was made fresh every 2 weeks (*n* = 6 active CORM-A1; 8 inactivated CORM-A1).

### Cell culture

Primary satellite cells were isolated and cultured from the entire hind limb of wild-type/control (WT) mice as previously described [19]. Cells were differentiated into myotubes using DMEM with 5% horse serum for 72 h. Cells were then infected with control (GFP) adenovirus (WT) or recombinant PGC-1α adenovirus (Ad-PGC-1α)) at multiplicity of infection of 10–30. Ad-PGC-1α contains PGC-1α expressed under control of the CMV promoter and has been described previously [20]; we have used it extensively, with robust induction of PGC-1α expression [17, 21, 22]. Cells were analyzed 48 h later.

### Evans blue injection and histological analysis

For Evans blue assay, mice were injected with 1% solution i.p. at a final concentration of 1% volume to body weight 16 h priors to sacrifice. Tissues were dissected and embedded in OCT compound (VWR) and flash-frozen. Evans blue was analyzed by fluorescence microscopy. Percentage area of Evans blue extravasation in the gastrocnemius was quantified, using ImageJ (NIH), by measuring the area of extravasation in at least ten ×200 images per condition. For purposes of quantifying central nuclei and fiber sizes, sections were stained with rabbit polyclonal anti-laminin antibody (Abcam, ab11575) in order to establish fiber boundaries. We used two different staining techniques to calculate centralized nuclei: (i) frozen sections were stained with anti-laminin and counterstained with DAPI; (ii) tissue was dehydrated and embedded in paraffin and sectioned before H&E staining. In either case, 20 images were taken at ×200 and number of centralized nuclei fibers to total fibers was counted in a blinded fashion. At least 500 fibers across sections from different animals were counted for each condition. For fiber sizes, all sizes were measured using ImageJ software, and 50 bins were created spanning the smallest to largest fiber size across the two comparison groups. For each group, the % of fibers in each bin was then calculated and plotted. Statistical comparison between groups was done using the Mann-Whitney *U* test.

### Serum creatine kinase assay

The blood was collected and serum isolated using heparin-coated collection tubes, either by heart puncture or cheek bleed. Blood was collected and spun down at maximum speed in heparinized tubes, after which serum was collected, flash-frozen, and stored at −80. Serum creatine kinase activity was then determined, simultaneously for all samples in a given experiment, with a Creatine Kinase-SL Assay Kit (Diagnostic Chemicals Limited).

### Real-time PCR

Total RNA was isolated from mouse tissue using the TRIzol (Invitrogen) reagent, while RNA from cultured cells were isolated using the Turbocapture (Qiagen) method. Samples for real-time PCR analyses were reverse transcribed (Applied Biosystems), and quantitative real-time PCRs were performed on the cDNAs in the presence of fluorescent dye (SYBR green; Bio-Rad). Relative expression levels were determined using the comparative cycle threshold method. All q-RT-PCR data were normalized to the average expression of three different housekeeping genes, TATA-binding protein (TBP), hypoxanthine phosphoribosyltransferase 1 (HPRT), and β-actin, as we have described previously [23]. Sequences are provided below:

Mouse

- HPRT F: GTTAAGCAGTACAGCCCCAAA
- R: AGGGCATATCCAACAACAAACTT
- TBP F: CCCTATCACTCCTGCCACACCAGC
- R: GTGCAATGGTCTTTAGGTCAAGTTTACAGCC
- B-ACTIN F: CCCTGTATGCCTCTGGTCGTACCAC
- R: GCCAGCCAGGTCCAGACGCAGGATG
- HMOX1 F: GGCGCACTCACCCTGAGCTGCTGG
- R: CCCAGAGCTGGGCAAGGCCATGG
- HMOX2 F: TCGGAGGGGGTAGATGAGTC
- R: GCTTCCTTGGTCCCTTCCTT
- Utrophin F: AGCCACCACATTTCGTTGGAA
- R: GACTTATCGAGAGAAAGTGAGGC
- VEGF-A F: GCCAGCACATAGAGAGAATGAGC
- R: CGGCTTGTCACATTTTTCTGG
- Cox5b F: CAGCTTGTAATGGGTTCCACAGT
- R: TTTTCTCACGCGGAGCTTTC
- PGC-1a F: AGCCGTGACCACTGACAACGAG
- R: GCTGCATGGTTCTGAGTGCTAAG

Human

- HPRT F: TGGACAGGACTGAACGTCTTG
- R: CCAGCAGGTCAGCAAAGAATTTA
- TBP F: GAGCCAAGAGTGAAGAACAGTC
- R: GCTCCCCACCATATTCTGAATCT
- B-actin F: GAGCGCGGCTACAGCTT
- R: TCCTTAATGTCACGCACGATTT
- HMOX1 F: GCAGAGGGTGATAGAAGAGGC
- R: GATGTTGAGCAGGAACGCAGT
- HMOX2 F: TCAGCGGAAGTGGAAACCTC
- R: AGAAGTCCTTGACAAACTGGGT

### Western blotting

Protein from gastrocnemius (100 μg) was run on a 4–20% polyacrylamide gel, transferred to nitrocellulose membrane, and blotted for heme-oxygenase I using an anti-HMOX1 antibody (ADI-SPA-896, Enzo Life Sciences), followed by goat anti-rabbit antibody conjugated to horseradish peroxidase (7074, Cell Signaling Technology Inc), followed by detection using ECL Reagent (Cell Signaling Technology Inc). As control, blot was stripped and probed with anti-pan-actin antibody (Cell Signaling Technology Inc). Blots were visualized and quantified with Biorad Image-Lab Software.

### Patient biopsies

Biopsies from patients were kindly obtained from the lab of Dr. Louis Kunkel. Biopsies were taken of the vastus lateralis of normal controls and latissimus dorsi of DMD muscle. The WT biopsies were from healthy male subjects ranging from 13 to 32 years of age. Biopsies from DMD patients were from 12 to 20 years of age with different genetic defects in the dystrophin gene.

### Statistical analysis

The data are presented as means ± SEM. Statistical analysis was performed with Student’s *t* test for all in vitro experiments and ANOVAs for all in vivo experiments. Fiber size comparisons were done using the Mann-Whitney *U* test. *P* values of <0.05 were considered statistically significant.

## Results

### Inhibition of VEGF signaling worsens dystrophy

**Fig. 1:**
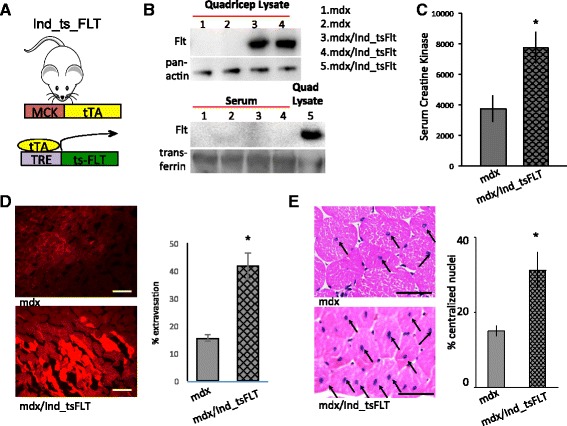
Inhibition of VEGF signaling worsens dystrophy. **a** Dual transgenic system to express both sFlt1 in the muscle only and only in absence of doxycycline. *MCK* muscle creatine kinase promoter, *tTA* tetracycline-suppressed transactivator, *TRE* tTA-responsive promoter. **b** Western blotting of sFlt1 demonstrating induction of sFlt1 in the muscle (*top*) and absence of spilling of sFlt1 into the circulation (*bottom*). **c**–**e** Serum creatine kinase levels (**c**), % Evans blue dye extravasation (**d**), and % central nuclei in gastrocnemius muscle (**e**) from *mdx* mice bearing the indicated transgenes. *Scale bar* = 50 μm. *n* = 8. *P < 0.05, Student’s *t* test. *Error bars* indicate ±SEM

We showed a number of years ago that PGC-1α powerfully induces angiogenesis in the skeletal muscle [21, 24]. The new vessels are functional, patent, non-leaky, and carry blood [25]. PGC-1α thus coordinately induces the consumption of fuel and oxygen (in mitochondria) with the delivery of fuel and oxygen (via blood vessels). Reduced blood flow and ischemia have been proposed to play an important role in DMD models, in part leading to the rationale for the recent use of the vasodilators sildenafil and tadalafil in clinical trials (with, thus far, mixed results). We therefore reasoned that perhaps, PGC-1α exerts its potent anti-dystrophic effects via dramatic increase in vascular density and blood flow. To test this notion, we first generated, in the *mdx* background, mice that express, in the muscle only and in a tetracycline-inducible manner, a locally secreted soluble VEGF receptor 1 (also known as soluble Fms-like tyrosine kinase 1, sFlt1). This VEGF “trap” neutralizes extracellular VEGF signaling and blocks angiogenesis. These mice, which we name Ind_tsFLT, were generated by breeding transgenic mice expressing tetracycline-inducible sFlt1, a kind gift of Eli Keshet [18]), with transgenic mice expressing muscle-specific expression of the tTA transactivator, also previously described [16], all in the mdx background (Fig. 1a). Thus, the mice express the VEGF trap only in the skeletal muscle and only in the absence of doxycycline. Mice were fed doxyclycine-containing chow throughout pregnancy, development, and weaning, to maintain the VEGF trap suppressed. At 5 weeks of age, mice were changed to normal chow, leading to robust expression of the VEGF trap in the skeletal muscle (Fig. 1b). Importantly, by virtue of a heparin-binding domain, the VEGF trap does not leak into the circulation or to other tissues (Fig. 1b), as originally reported [18]. Littermate mice that did not contain the sFLT transgene were used as controls and were similarly maintained on doxy-chow and released to normal chow at 5 weeks, thus controlling for any effects of doxycycline chow.

*Mdx* mice undergo cycles of muscle degeneration and regeneration, most marked during the first 3–10 weeks of life. Muscle degeneration can be detected during this period in a number of fashions, including accumulation of muscle creatine kinase (CK) in serum, and extravasation of Evans blue dye, which normally remains intravascular in the absence of tissue damage. Strikingly, induction of the VEGF trap in the skeletal muscle of *mdx* mice led to a near doubling of serum CK levels (Fig. 1c). This was accompanied by marked increase in EBD extravasation (Fig. 1d). Following muscle degeneration, cycles of regeneration are driven by the activation of resident progenitor cells and their fusion with degenerating myofibers. This process can be detected by the appearance and accumulation of central nuclei, which otherwise normally reside in the periphery of fibers, as well as the accumulation of small regenerated myofibers. Consistent with the increase in muscle damage, induction of the VEGF trap in the skeletal muscle of *mdx* mice markedly increased the accumulation of central nuclei (Fig. 1e). These data demonstrate that suppression of VEGF signaling in *mdx* mice markedly worsens muscle dystrophy in this model, consistent with the notion that impaired vascular homeostasis worsens dystrophy.

### PGC-1α protects against dystrophy independently of angiogenesis

**Fig. 2:**
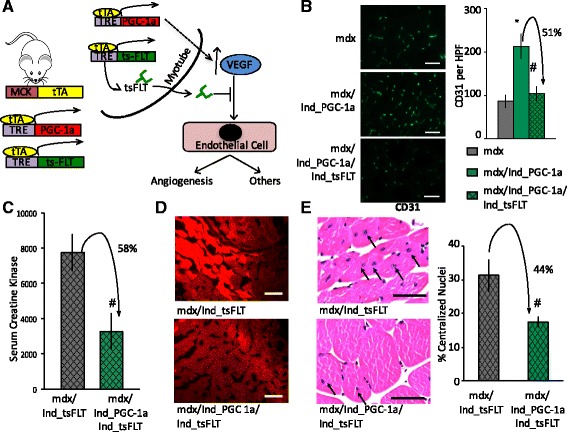
PGC-1α protects against dystrophy independently of angiogenesis. **a** Tri-transgenic system to express both PGC-1α and sFlt1 in the muscle only and only in absence of doxycycline. *MCK* muscle creatine kinase promoter, *tTA* tetracycline-suppressed transactivator, *TRE* tTA-responsive promoter. **b** PGC-1α-induced angiogenesis is blocked in the presence of sFlt1: capillary density, identified by CD31 staining, in muscle cross sections from *mdx* mice bearing the indicated transgenes; quantification is on the *right*. **c**–**e** Despite absence of angiogenesis, PGC-1α protects against dystrophy: serum creatine kinase levels (**c**), sample Evans blue staining in diaphragm (**d**), and % central nuclei in gastrocnemius muscle (**e**) from *mdx* mice bearing the indicated transgenes. *n* = 8. *P < 0.05 versus mdx alone, ^#^ *P* < 0.05 versus mdx/Ind_PGC-1α, Student’s *t* test. *Scale bar* = 50 μm. *Error bars* indicate ±SEM

Can suppression of VEGF signaling abrogate the ability of PGC-1α to improve muscle dystrophy? To test this, we used a triple transgenic model, in the background of the *mdx* model, whereby both PGC-1α and the VEGF trap are simultaneously induced in the skeletal muscle upon removal of doxycycline (Fig. 2a). This was achieved by crossing Ind_tsFlt mice with mice containing a third transgene encoding for tetracycline-inducible PGC-1α, a kind gift from Dr. Dan Kelly [16]. Therefore, in these triple transgenic mice, the VEGF trap and PGC-1α are simultaneously induced, only in the skeletal muscle and only in the absence of doxycycline. Induction of PGC-1α alone (without the VEGF trap) led to a robust induction of vascular density, which was completely abrogated with co-induction of the VEGF trap (Fig. 2b), as we have shown before in a non-dystrophic context [17], thus demonstrating the effectiveness of suppression of angiogenesis in this model. We therefore used this system to test the hypothesis that PGC-1α improves muscle dystrophy by virtue of stimulating vascular density. If true, then induction of PGC-1α in the context of simultaneously induced VEGF trap should show no improvement compared to the VEGF trap alone. Strikingly, however, despite the complete block in angiogenesis, the induction of PGC-1α was still able to protect against dystrophy in the presence of the VEGF trap, as shown by reduction of circulating CK (Fig. 2c), reduced extravasation of EB dye (Fig. 2d), and reduced accumulation of central nuclei (Fig. 2e). Thus, these data show that suppression of VEGF signaling in *mdx* mice does not prevent PGC-1α from protecting against muscle dystrophy in this model.

### HO-1 expression is reduced in dystrophic muscle and is rescued by PGC-1α

How does PGC-1α protect against muscle dystrophy, if not via utrophin, via NMJ induction, or via increases in vascular density? We reasoned that identifying other pathways induced by PGC-1α may point to novel pathways involved in protection against dystrophy. In search of genes induced by PGC-1α that may have protective roles in the skeletal muscle, our attention was caught by *Hmox1*, the gene encoding for heme oxygenase (HO-1), as a gene of interest. HO-1 degrades the cyclic prosthetic group heme, liberating ferrous iron and yielding biliverdin and gaseous carbon monoxide (CO) in the process. Heme is abundant in the skeletal muscle, present for example in myoglobin and mitochondrial cytochromes. A growing literature supports a protective role for HO-1 in various contexts, though it remains unclear to what extent these benefits accrue from the degradation of toxic heme, the generation of anti-oxidant biliverdin, and/or protective effects of CO [26–28]. We therefore decided to focus attention on HO-1. Interestingly, the Kunkel lab recently identified small molecule inducers of HO-1 as protective in a zebrafish model of DMD, although subsequent experiments raised into question if protection involved increasing HO-1, as originally proposed [29, 30].

**Fig. 3:**
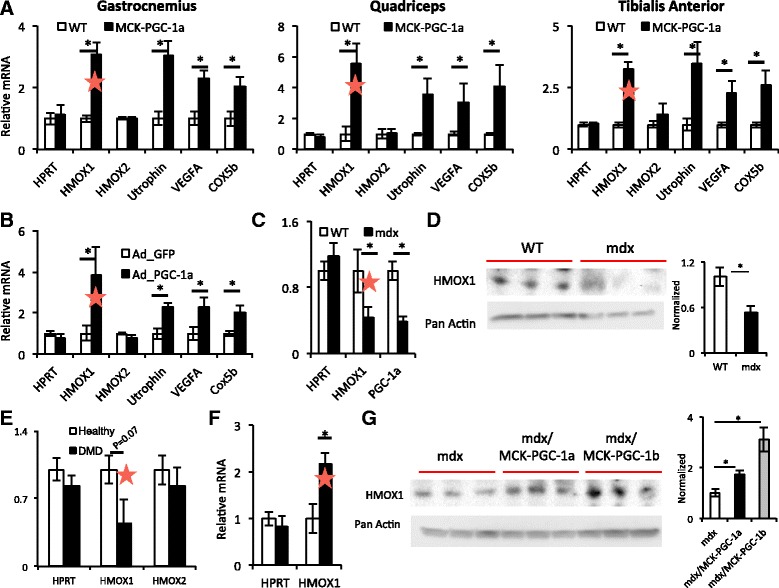
HO-1 expression is reduced in dystrophic muscle, and PGC-1α rescues HO-1 expression. **a** Relative gene expression of the indicated genes in the indicated muscle beds from transgenic mice expressing PGC-1α in the skeletal muscle (MCK-PGC-1a), versus controls. **b** Relative gene expression of the indicated genes in myotubes expressing adenovirus-driven PGC-1α (ad_PGC-1a), versus GFP controls. **c**, **d** Relative gene expression (**c**) and protein expression (**d**) of HO-1 in the muscle from *mdx* mice, versus controls. Quantification is shown on the *right*. **e** Relative gene expression of the indicated genes in the muscle from patients with DMD, versus matched controls. *N* = 3 per group. **f**, **g** Relative gene expression (**f**) and protein expression (**g**) of HO-1 in the muscle from *mdx/*MCK-PGC1a and *mdx*/MCK-PGC-1b transgenic mice, versus *mdx* alone. Quantification of protein expression is shown on the *right. n* = 5. *Red bars* highlight HMOX1 data. *P < 0.05, Student’s *t* test. *Error bars* indicate ±SEM

Consistent with previous reports, we find induction of mitochondrial (cox5b) and angiogenic (VEGFA) genes, and utrophin, in quadriceps, gastrocnemius, and tibialis anterior, of mice that transgenically express PGC-1α in the skeletal muscle under control of the muscle creatine kinase promoter (MCK-PGC-1α mice) (Fig. 3a). In addition, we noted a strong threefold induction of *Hmox1*. Adenoviral delivery of PGC-1α to differentiated primary myotubes induced *Hmox1* fourfold (Fig. 3b), indicating that the induction of *Hmox1* by PGC-1α is cell autonomous. To assess the role for HO-1 in muscle dystrophy, we first tested its expression in the muscle from *mdx* mice. As shown in Fig. 3c, d, both transcript and protein expression of HO-1 were depressed more than 50% in the muscle from these mice, compared to age-matched, sex-matched, C57/BL10 mice, purchased from the same vendor and bred and maintained in the same mouse facility and suite as the mdx mice. Evaluation of human muscle biopsies revealed a strong trend for depressed expression of *HO-1* (Fig. 3e, *P* < 0.07) (although these data must be interpreted with caution, in light of small number of samples and variability of biopsy site). Transgenic expression of PGC-1α in the *mdx* background reversed this inhibition, inducing *Hmox1* transcript and HO-1 protein more than twofold compared to littermate controls not expressing the transgene (Fig. 3f, g). Transgenic expression of PGC-1β in the skeletal muscle also induced HO-1 more than twofold in *mdx* mice compared to littermate controls not expressing the transgene (Fig. 3g). These data indicate that HO-1 expression is depressed in muscle dystrophy and that induction of PGC-1α can reverse the repression of HO-1.

### Increasing HO-1 expression ameliorates muscle damage, while decreased HO-1 expression exacerbates muscle damage in mdx mice

**Fig. 4:**
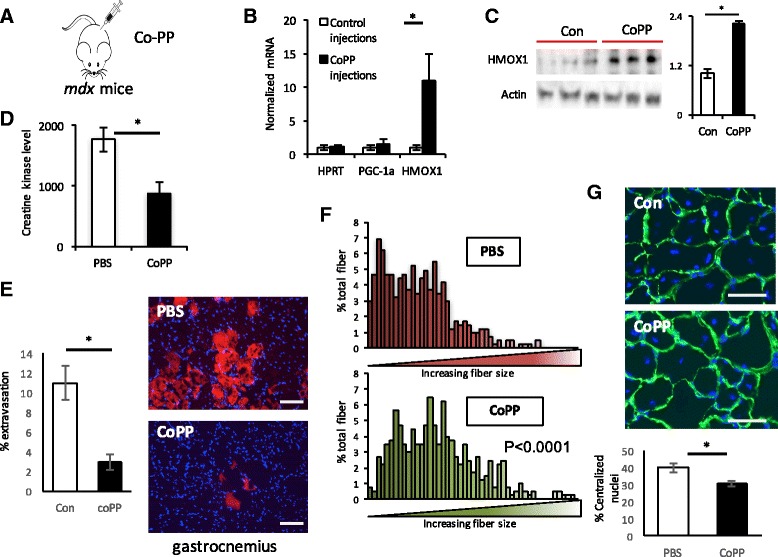
Induction of HO-1 protects against dystrophy. **a** Model. **b**, **c** Relative gene expression (**b**) and protein expression (**c**) of HO-1 in the muscle from *mdx* mice treated with Co-PP, versus vehicle. Quantification is shown on the *right* in **c. d–g** Serum creatine kinase levels (**d**), and % Evans blue extravasation (**e**), fiber size distribution (**f**), and % central nuclei (**g**) in gastrocnemius muscle from *mdx* mice treated with Co-PP, versus vehicle. *n* = 8. *P < 0.05, Student’s *t* test. *Scale bar* = 50 μm. *Error bars* indicate ±SEM

In light of the known beneficial effects of PGC-1α in *mdx* mice, the data above suggested that induction of HO-1 may improve dystrophy in this model. To test this notion, we treated *mdx* mice with cobalt protoporphyrin (Co-PP), a well-established and powerful inducer of HO-1 expression [31]. Treatment of *mdx* mice with injections of Co-PP three times a week for 4 weeks led to robust induction of HO-1 mRNA and protein expression measured in the gastrocnemius muscle (Fig. 4a–c). Strikingly, accumulation of CK in the serum of *mdx* mice was reduced by 50% (Fig. 4d) by treatment with Co-PP. This was accompanied by visible inhibition of Evans blue dye extravasation in the diaphragm and gastrocnemius (Fig. 4e). Consistent with the inhibition of muscle damage, treatment of *mdx* mice with Co-PP preserved larger myofiber size (Fig. 4f) and markedly reduced the accumulation of central nuclei (Fig. 4g). These data demonstrate that treatment with Co-PP and induction of HO-1 in *mdx* mice markedly improves muscle dystrophy in this model.

**Fig. 5:**
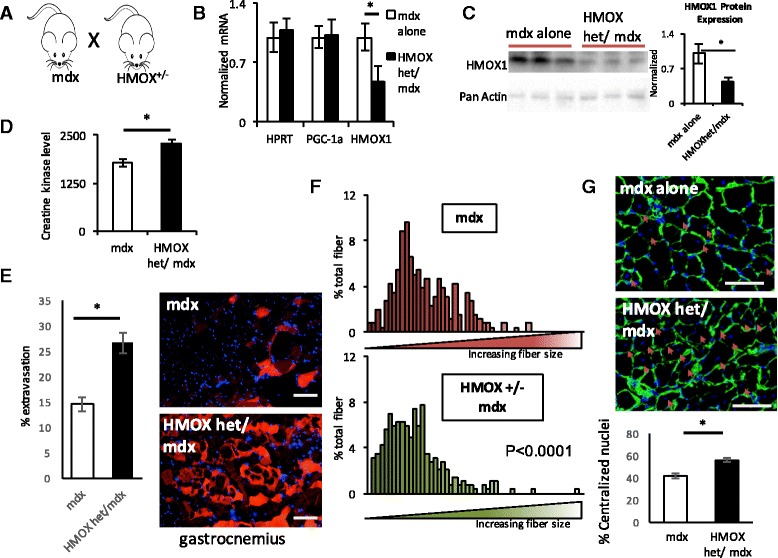
Suppression of HMOX1 worsens muscle dystrophy. **a** *mdx* mice were crossed with HMOX1 heterozygotes. **b**, **c** Relative gene expression (**b**) and protein expression (**c**) of HMOX1 in the muscle from *mdx* and *mdx hmox* ^*+/−*^ mice. Quantification is shown on the *right* in **c. d–g** Serum creatine kinase levels (**d**), and % Evans blue extravasation (**e**), fiber size distribution (**f**), and % central nuclei (**g**) in gastrocnemius muscle from *mdx* versus *mdx hmox* ^*+/−*^ mice. *n* = 8. *P < 0.05, Student’s *t* test. *Scale bar* = 50 μm. *Error bars* indicate ±SEM

The data above indicate that induction of HO-1 is sufficient to protect against muscle dystrophy. To test if HO-1 is also necessary for endogenous adaptation to dystrophy, we crossed mice heterozygous null at the *Hmox1* locus into the *mdx* background (Fig. 5a). Mice lacking HO-1 have been described [32]. Homozygous mice are sometimes viable but display numerous phenotypes, including anemia, abnormal iron storage and metabolism, and chronic inflammation. On the other hand, heterozygous mice are largely normal at baseline. As shown in Fig. 5b, c, the expression of HO-1 mRNA and protein is decreased by 50% in heterozygous mice, indicating haploinsufficiency at this locus. The mice are thus useful to test the role of HO-1 in adaptation to the *mdx* background. No abnormalities were seen in the muscle from *Hmox1* ^+/−^ mice at baseline. On the other hand, as shown in Fig. 5d, accumulation of CK in the serum was significantly worsened in *Hmox1* ^*+/−*^ */mdx* mice, compared to *mdx* alone. This was accompanied by visibly more Evans blue dye extravasation in the diaphragm and gastrocnemius (Fig. 5e). Evidence of compensatory increases in regeneration was seen with significantly smaller fiber sizes (Fig. 5f) and significantly more central nuclei (Fig. 5g). Haploinsufficiency at the *Hmox1* locus thus worsens dystrophy in *mdx* mice, indicating that HO-1 is required for adaptations to the *mdx* background.

### Carbon monoxide treatment ameliorates muscle damage in mdx mice

**Fig. 6:**
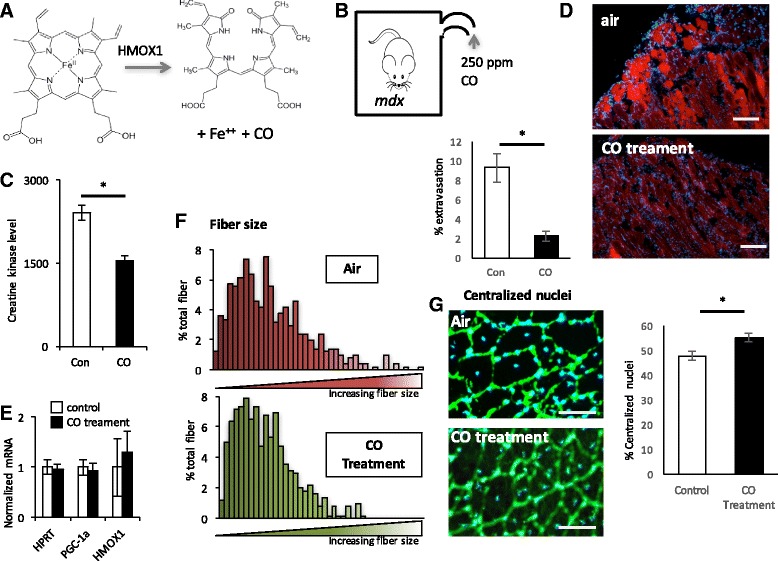
Inhaled low-dose carbon monoxide protects against dystrophy. **a** Schematic of HO-1 enzymatic action. **b** Mice were placed in chambers containing 250 ppm CO for 1 h per day. **c**, **d** Serum creatine kinase (**c**) and sample Evans blue staining in diaphragm and % Evans blue extravasation in gastrocnemius (**d**) in *mdx* mice exposed to CO versus air alone. **e** Relative expression of the indicated genes in gastrocnemius from *mdx* mice exposed to CO versus air alone. **f**, **g** Fiber size distribution in gastrocnemius (**f**) and % central nuclei in gastrocnemius muscle (**g**) from *mdx* mice exposed to CO versus air alone. *n* = 8. *P < 0.05, Student’s *t* test. *Scale bar* = 50 μm. *Error bars* indicate ±SEM

By what mechanism does HO-1 improve dystrophy? HO-1 degrades the cyclic prosthetic group heme, liberating ferrous iron and yielding biliverdin and CO gas in the process (Fig. 6a). The benefits of HO-1 could in theory accrue from the degradation of toxic heme, the generation of anti-oxidant biliverdin, and/or protective effects of CO itself. We therefore tested if delivery of CO alone to *mdx* mice could confer protection against dystrophy. We achieved delivery of CO by two methods: first, inhalation of low-dose CO (250 ppm, 0.025%) and, second, by using CO-releasing molecules (CO-RMs). CORM-A1 is a water soluble CO-releasing molecule with a relatively long half-life (21 min at 37 °C, pH 7.4 [33]). Exposure of *mdx* mice to ambient low-dose CO for 1 h/day for 4 weeks led to a dramatic improvement in dystrophy, evidenced by marked improvement of Evans blue extravasation in diaphragm, and a 40% reduction in circulating creatine kinase levels (Fig. 6b–d). *Hmox1* expression was not induced by CO (Fig. 6e), consistent with mechanistic activity downstream of HO-1. Interestingly, myofiber sizes were not altered with CO exposure (Fig. 6f). In addition, central nuclei were slightly increased (Fig. 6g), suggesting that CO may also affect myoprogenitor proliferation or differentiation.

**Fig. 7:**
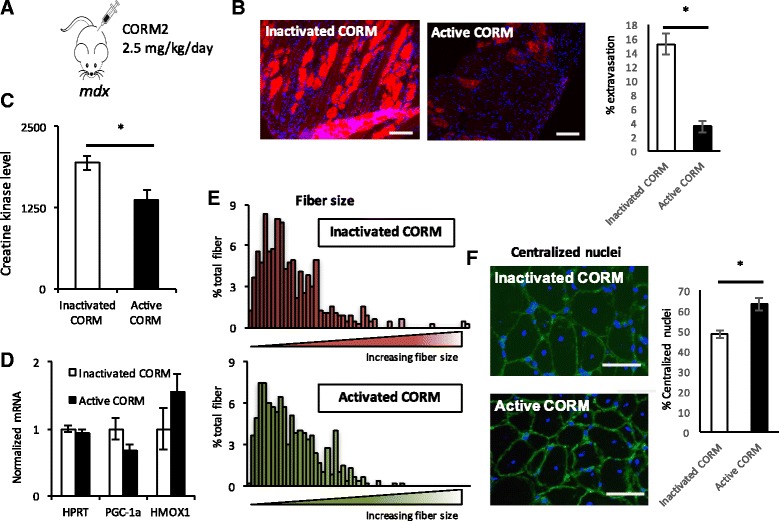
Delivery of carbon monoxide via small molecules protects against dystrophy. **a** Mice were treated daily with 2.5 mg/kg CORM-A1. **b**, **c** Sample Evans blue staining in diaphragm and % Evans blue extravasation in gastrocnemius (**b**) and serum creatine kinase (**c**) in *mdx* mice treated with CORM-A1 versus vehicle. **d** Relative expression of the indicated genes in gastrocnemius from *mdx* mice treated with CORM-A1 versus vehicle. **e**, **f** Fiber size distribution (**e**) and % central nuclei (**f**) in gastrocnemius muscle from *mdx* mice treated with CORM-A1 versus vehicle. *n* = 8. *P < 0.05, Student’s *t* test. *Scale bar* = 50 μm. *Error bars* indicate ±SEM

Treatment of *mdx* mice with daily injections of CORM-A1 for 4 weeks recapitulated all of the findings seen with inhaled CO, including marked improvements in dystrophy, with a dramatic reduction of Evans blue extravasation, and reduced circulating CK levels (Fig. 7a–c), unaltered expression of *Hmox1* (Fig. 7d), unaltered myofiber sizes (Fig. 7e), and slight increase in central myonuclei (Fig. 7f).

Together, these data indicate that delivery of CO, either via inhalation or via pharmaceutical delivery, strongly improves indices of muscle dystrophy.

## Discussion

We have leveraged here the transcriptional coactivator PGC-1α to identify a novel pathway, involving the heme oxygenase enzyme and its enzymatic product carbon monoxide, as potent protective agents against dystrophy. One of the more appealing and transformative aspects of the work is its potential imminent translatability to the clinical setting. CO has an understandably nefarious public image, but the toxicity of CO occurs at doses orders of magnitude higher than those used here. Indeed, CO is produced endogenously by HO-1 under normal physiology. The safety of CO in humans has been demonstrated in phase I dose escalation with no evidence of toxicity (clinicaltrials.gov NCT01050933, Otterbein personal communication, and [26]). Inhaled CO was also found to be safe in patients with COPD [34]. There are currently ongoing clinical trials of inhaled CO for treatment of adult and neonatal pulmonary hypertension (NCT01523548 and NCT01818843, respectively), ARDS (NCT02425579), post-operative ileus (NCT01050712), idiopathic pulmonary fibrosis (NCT01214187), and cardiac mitochondrial dysfunction (NCT01727167), extensively underscoring the accepted toxicity profile of CO. Importantly, in our experiments and in all of the above studies, inhaled CO is delivered only in intermittent doses, in our case 1 h a day. Translation to the clinic is therefore pragmatically feasible. Patients with DMD often have reduced respiratory capacity, in which case, the dose of delivery CO could be adjusted by measuring diffusing capacity of the lung for carbon monoxide (DLCO), a widely used clinical test. Alternatively, CO-releasing molecules (CO-RMs) may also serve as bioavailable alternatives to inhaled CO, which would both facilitate delivery and allay fears of inhaling a gaseous therapeutic. Our work thus suggests that delivery of CO, either by inhalation of by CO-RMs, could be ready for clinical trials, and/or trials in large animals, to test for efficacy in the treatment of Duchenne muscular dystrophy, and perhaps other dystrophies as well.

How does heme oxygenase protect against dystrophy? Heme oxygenase degrades the cyclic prosthetic group heme, liberating ferrous iron and yielding biliverdin and gaseous CO (Fig. 3a). The protective effects of HO-1 could therefore in principle stem from reductions of toxic heme or from beneficial effects elicited by biliverdin, iron, or CO. Mechanistically, we show here that CO alone is sufficient to protect against muscle dystrophy. Additional involvement by the other enzymatic products is also possible, as is suggested for example by the observation that treatment with CO or CORM both led to protection from damage, as determined by CK and EBD leak, but did not lead to larger fibers, in contrast to treatment with HO-1 activators.

How CO itself protects against dystrophy is also of interest and will be the subject of future studies. One possibility is the observation that CO can, in some contexts, promote the formation of NO [26]. NO has been espoused as beneficial in models of dystrophy, perhaps in part via vasodilation and improved local ischemia [3, 4, 35]. Exposure to CO can also change cellular bioenergetics and increase oxygen consumption [36, 37] and can increase mitochondrial biogenesis in the muscle from humans and animals [38, 39]. CO-induced mitochondrial biogenesis may thus contribute to protection against dystrophy and if so may constitute a positive feedback loop with PGC-1α expression.

Although we have focused our attention here on heme oxygenase as a novel therapeutic opportunity, the anti-dystrophy protection that is afforded by PGC-1α is likely multifactorial and is unlikely limited only to the induction of heme oxygenase. Because complete deletion of *Hmox1* has diffuse effects, testing the relative contribution of the HO-1 system to PGC-1α-mediated protection will require introducing muscle-specific deletion of *Hmox1* into transgenic PGC-1α mice in the *mdx* background.

## Conclusions

In summary, our work highlights a new and actionable approach to the treatment of patients with DMD, including the appealing possibility that low-dose inhalation of CO could provide a novel and simple therapy for this devastating disease.

## Abbreviations

CO: Carbon monoxide
CORM-A1: CO-releasing molecule A1
DMD: Duchenne muscular dystrophy
HO: Heme oxygenase
MCK: Muscle creatine kinase
mdx: Dystrophin-deficient mice
NMJ: Neuromuscular junction
nNOS: Nitric oxide synthase
PGC-1α: Peroxisome proliferator-activated receptor gamma coactivator 1-α
PGC-1β: Peroxisome proliferator-activated receptor gamma coactivator 1-β
TRE: tet-response element promoter
tTA: Tetracycline-dependent activator
VEGF: Vascular endothelial growth factor
WT: Wild-type/control
